# A comparison study of anxiety in children undergoing brain MRI vs adults undergoing brain MRI vs children undergoing an electroencephalogram

**DOI:** 10.1371/journal.pone.0211552

**Published:** 2019-03-07

**Authors:** Charlotte Jaite, Viola Kappel, Adriane Napp, Marcus Sommer, Gerd Diederichs, Bernhard Weschke, Birgit Spors, Arpad von Moers, Ulrike Lehmkuhl, Christian J. Bachmann

**Affiliations:** 1 Department of Child and Adolescent Psychiatry, Charité - Universitätsmedizin Berlin, Berlin, Germany; 2 Department of Radiology, Charité - Universitätsmedizin Berlin, Berlin, Germany; 3 GeRN-Gesellschaft für Radiologie und Nuklearmedizin GbR, Wilhelmshaven, Germany; 4 Department of Pediatric Neurology, Charité - Universitätsmedizin Berlin, Berlin, Germany; 5 Department of Pediatrics, DRK Kliniken Berlin Westend, Berlin, Germany; 6 Department of Child and Adolescent Psychiatry, LVR-Klinikum Düsseldorf/ Kliniken der Heinrich-Heine-Universität Düsseldorf, Düsseldorf, Germany; Southwest University, CHINA

## Abstract

**Background:**

Magnetic resonance imaging (MRI) of the brain in children and adolescents is a well-established method in both clinical practice and in neuroscientific research. This practice is sometimes viewed critically, as MRI scans might expose minors (e.g. through scan-associated fears) to more than the legally permissible “minimal burden”. While there is evidence that a significant portion of adults undergoing brain MRI scans experience anxiety, data on anxiety in children and adolescents undergoing brain MRI scans is rare. This study therefore aimed to examine the prevalence and level of anxiety in children and adolescents who had MRI scans of the brain, and to compare the results to adults undergoing brain MRI scans, and to children and adolescents undergoing electroencephalography (EEG; which is usually regarded a “minimal burden”).

**Method:**

Participants were 57 children and adolescents who had a brain MRI scan (MRI-C; mean age 12.9 years), 28 adults who had a brain MRI scan (MRI-A; mean age 43.7 years), and 66 children and adolescents undergoing EEG (EEG-C; mean age 12.9 years). Anxiety was assessed on the subjective (situational anxiety) and on the physiological level (arousal), before and after the respective examination.

**Results:**

More than 98% of children and adolescents reported no or only minimal fear during the MRI scan. Both pre- and post-examination, the MRI-C and the MRI-A groups did not differ significantly with respect to situational anxiety (*p* = 0.262 and *p* = 0.374, respectively), and to physiological arousal (*p* = 0.050, *p* = 0.472). Between the MRI-C and the EEG-C group, there were also no significant differences in terms of situational anxiety (*p* = 0.525, *p =* 0.875), or physiological arousal (*p* = 0.535, *p* = 0.189). Prior MRI experience did not significantly influence subjective or physiological anxiety parameters.

**Conclusions:**

In this study, children and adolescents undergoing a brain MRI scan did not experience significantly more anxiety than those undergoing an EEG, or adults undergoing MRI scanning. Therefore, a general exclusion of minors from MRI research studies does not appear reasonable.

## Introduction

The last three decades have seen a steady rise in the importance of magnetic resonance imaging (MRI) and in the number of MRI studies performed both for clinical purposes and for medical research [1, 2].

However, 10% to 37% of persons undergoing MRI, reportedly suffer from unpleasant effects of the procedure, particularly anxiety of various kinds, including claustrophobia [3–5]. Some authors have expressed the view that anxiety during MRI procedures may be even more pronounced among children and adolescents than among adults, because adults have more experience with medical procedures and their cognitive compensatory strategies are more fully developed [6]. In some places, this has led to controversies whether children and adolescents are allowed to participate in MRI scans that are performed solely for research purposes (e.g. fMRI paradigms in studies exploring the neural basis of ADHD), as MRI scans might expose minors (e.g. through scan-associated fears) to more than the legally permissible “minimal burden” [7, 8].

To date, the research literature regarding anxiety in children and adolescents undergoing MRI scans is scarce. Westra et al. [9] and Haddad et al. [10], in their respective studies, concluded that MRI caused a negligible amount of stress, if any, in the vast majority of the children and adolescents who underwent it; only a few of their subjects perceived the procedure as more unpleasant than blood drawing. In contrast, Tyc et al. [11] and Marschall et al. [3] yielded different findings: In their studies, about 30% of children and adolescents undergoing MRI reported anxiety. Interestingly, in the Tyc et al. study, the greatest anxiety was not produced by the MRI scan per se, but rather by the associated insertion of an intravenous catheter. However, the significant anxiety rates in both studies might also be explained by these studies having been conducted more than 20 years ago. At that time, MRI scanners made more noise during scans, gantries were narrower, and scans took considerably longer, thus potentially inducing more anxiety [12].

Only three studies to date have included a direct comparison of the anxiety experienced by children and adolescents undergoing MRI with that of adults. Shechner et al. [13] and Thomason [14] found that children had the same emotional responses to MRI as adults, and that some children even gained more pleasure from the experience than adults did. Shechner et al. also found that anxious children displayed no more anxiety during MRI procedures than their non-anxious counterparts did. In three further studies [3, 9, 11], no association was found between young age of the subject and increased anxiety during MRI. However, in a study with a comparably small sample, Galván et al. found that children and adolescents had more anxiety during MRI than adults did [15].

In view of the scant available evidence, this study aimed to compare anxiety levels in children and adolescents undergoing MRI scanning of the brain (bMRI) with (1) adults having a bMRI, and (2) children and adolescents undergoing electroencephalography (EEG; which is usually regarded a “minimal burden”).

## Materials and methods

### Ethics committee approval

This study was approved by the Ethics Committee of the Charité-Universitätsmedizin Berlin, Germany (EA2/036/11). Written informed consent was obtained from all adult participants, and from the parents or legal guardians of all participating minors.

### Inclusion and exclusion criteria

Inclusion criteria for all three groups were a medical indication for the examination in question, and an IQ ≥85. The MRI-C group consisted of children and adolescents (age range: 8;0 to 17;11 years) who underwent brain MRI scanning. Comparison groups consisted of a random sample of children and adolescents, likewise aged 8;0 to 17;11 years, who underwent EEG recording (the EEG-C group), and a random sample of adult patients, aged 18;0 to 64;11 years, who underwent bMRI (the MRI-A group). Exclusion criteria were the usual contraindications for MRI (e.g., cardiac pacemaker, defibrillator, metallic fragments in biologically sensitive regions), emergency/high-urgency MRI scans or EEG recordings, severe neurological, somatic, or psychiatric disorders, and a diagnosed anxiety disorder.

### MRI scans and EEG recordings procedure

The MRI scans were carried out at the Department of Radiology, Charité - Universitätsmedizin Berlin, and at the Department of Radiology, DRK Kliniken Berlin Westend (MR scanners: Siemens 1.5 T Magnetom Symphony, Siemens 1.5 T Magnetom Avanto, and Philips 1.5 T Gyroscan Intera Achieva). Only MRI scans in which no contrast medium was given (i.e., scans for which no intravenous access needed to be obtained) were evaluated. The EEG recordings were performed at the Department of Paediatric Neurology, Charité - Universitätsmedizin Berlin, using a 10–20 setting with 21 channels on a Nihon Kohden Neurofax EEG-9210G system.

### Measured variables

Measurements took place at three time points: at baseline (T0), as well as immediately before (T1), and immediately after (T2) the MRI scan or EEG recording.

#### Measurements at T0

Within fourteen days before the diagnostic procedure, the participants’ IQ was measured, using the *Culture Fair Test 1 (CFT-1)* [16] for children up to an age of 9;5 years, and the *Culture Fair Test 20*, *revised version (CFT-20-R)* [17] for all participants aged 9;6 years and older. The CFT is a well-established IQ measure that consists of five (CFT-1) and four subtests (CFT-20), respectively, and has good psychometric properties [16, 17].

Additionally, the possible presence of an anxiety disorder was examined, employing the *Child Behavior Checklist 4–18 (CBCL/4-18)* [18] for children and adolescents, and the *Symptom Checklist 90-Revised (SCL-90-R)* [19] for adults. The CBCL/4-18 is an 86-item parent-report screening instrument, measuring both competencies and psycho-somatic problems in children and adolescents (age range: 4 to 18 years). It consists of three competency subscales and eight problem subscales, which can be transformed into an externalizing, an internalizing, and a total problem score. Both reliability and internal consistency of the CBCL/4-18 is good to very good [18]. For the purpose of this study, we used the anxiety/depression subscale, with a cut-off value of >74 for anxiety disorders.

The SCL-90-R is a 90-item screening instrument with good psychometric properties that assesses nine psychopathology symptom dimensions, and provides three global distress indices, which can be compared to nonpatient, outpatient or inpatient norms [18]. In order to determine the presence of an anxiety disorder, we used the anxiety subscale, with a cut-off point of > 74.

#### Measurements at T1 and T2

In children, the *State-Trait Anxiety Inventory for Children* (STAIC; [20]), the *Patient Experience Questionnaire* (PEQ; [21]), and the *Physiological Hyperarousal Scale for Children* (PH-C; [22]) were applied. For adolescents and adults, the *State-Trait Anxiety Inventory* (STAI; [23]), the *Patient Experience Questionnaire* (PEQ; [21]), and the PH-C were used.

The STAI and the STAIC are self-report measures for assessing state and trait anxiety with two 20-item scales. Both STAI and STAIC are similar in conception and structure and have robust psychometric properties [20, 23].

The PH-C is a self-report questionnaire and consists of 18-items to measure physiological hyperarousal, defined as physical manifestations of autonomic arousal. The scale was originally developed for children, but can also be used in other age groups. It has good psychometric properties [22].

The PEQ is a self-report instrument that evaluates retrospectively patients’ experiences during the MRI scan with the four subscales “claustrophobia and restricted mobility”, “lack of information/clarity”, “procedural disturbing factors and negative thoughts”, and “physical discomfort2. We also used the PEQ in the EEG-C sample to assess patients’ experiences during the EEG recording. Details of the psychometric properties are not available yet [21].

Heart rate and blood pressure were measured, using a fully automated Sanitas SBM 03 blood pressure meter.

### Statistical analysis

All statistical analyses were performed using IBM SPSS Statistics 25. To test for differences between groups, chi-square tests, t-tests for independent samples, and one-way ANOVAs were conducted. Bonferroni post hoc tests for pairwise comparisons were employed to adjust for multiple comparisons.

Differences in anxiety parameters between groups were tested with univariate covariance analysis (ANCOVA). Because of intergroup differences in trait anxiety at baseline (*p* = <0.001) (Table 1), this variable was considered as a covariate. Due to developmental reasons, heart rate and blood pressure are not comparable between children and adults. Therefore, changes in heart rate and blood pressure from T1 to T2 in the MRI-C and MRI-A groups were evaluated via t-tests for dependent samples.

**Table 1:** Sample characteristics.

	MRI-C *M ± SD*, *n* (%)	EEG-C *M ± SD*, *n* (%)	MRI-A *M ± SD*, *n* (%)	ANOVA / T-Test / x^2^-Test		
				*F / t / x^2^*	*df*	*p*
Characteristics						
Age (in years)	12.7 ± 3.1	12.3 ± 2.8	43.7 ± 14.4	232.71	2	<0.001
Sex (% males)	25 (43.9%)	34 (51.5)	9 (32.1)	3.032	2	0.220
IQ	101.8 ± 11.8	102.8 ± 13.2	98.5 ± 12.2	1.15	2	0.319
CBCL, anxiety/depression scale	56.9 ± 7.8	54.6 ± 6.1	-	-1.891	113	0.071
SCL-90-R, anxiety scale	-	-	52.9 ± 11.0	-	-	-
STAI(C)-T, t value (T1)	47.0 ± 12.6	44.7 ± 9.2	61.3 ± 10.2	23.45	2	<0.001
Procedure duration (in min.)	11.3 ± 2.2	11.1 ± 2.0	12.6 ± 3.1	2.14	2	<0.001
Prior experience of diagnostic procedure						
- None - At least one	23 (40.4) 34 (59.6)	34 (51.5) 32 (48.5)	15 (53.6) 13 (46.4)	2.006	2	0.367

CBCL: Child Behavior Checklist, SCL-90-R: Symptom Checklist 90-R, PH-C: Physical Hyperarousal Scale for Children, STAI-S: State-Trait-Anxiety Inventory, state anxiety scale, STAIC-S: State-Trait-Anxiety Inventory for Children, state anxiety scale.

In addition, subgroup analyses (children and adolescents who underwent a single MRI scan vs. those who underwent multiple MRI scans) were carried out, using t-tests for independent samples. Multiple regression analyses were computed to predict the anxiety experience. The following variables were examined as possible predictors: Age, sex, IQ, trait anxiety score, number of prior MRI scans, and anxiety about MRI scan findings. The significance level was set at *p*<0.05.

## Results

### Sample characteristics

The overall sample population consisted of 182 subjects, 31 (17.0%) of whom had an IQ <85 and were therefore excluded from further analysis. Consequently, the statistical analyses were carried out on the following numbers of subjects in the three groups: 66 in the EEG-C group, 57 in the MRI-C group, and 28 in the MRI-A group.

There were no significant group differences in sex distribution (*p* = 0.220), IQ (*p* = 0.319), or the number of prior MRI or EEG studies (*p* = 0.367). The subjects in the MRI-A group had significantly higher anxiety trait scores (STAI(C)-T) at time point T0 (*p*<0.001) and longer procedure durations than the subjects in the other two groups (*p*<0.001). Further features of the three groups and the anxiety parameters before and after MRI scanning and EEG recording, respectively, are shown in Tables 1 and 2.

**Table 2:** Anxiety parameters before (T1) and after (T2) the diagnostic procedure (MRI or EEG).

	MRI-C *M ± SD*	EEG-C *M ± SD*	MRI-A *M ± SD*	ANCOVA MRI-C vs. MRI-A			ANCOVA MRI-C vs. EEG-C		
				*F*	*df*	*p*	*F*	*df*	*p*
Parameters									
STAI(C)-S, t value (T1)	50.6 ± 11.6	48.8 ± 8.3	58.9. ± 11.2	1.287	1	0.262	0.407	1	0.525
STAI(C)-S, t value (T2)	46.6 ± 10.6	45.4 ± 9.4	54.0 ± 10.3	0.799	1	0.374	0.025	1	0.875
PH-C, mean score (T1)	1.4 ± 0.4	1.4 ± 0.3	1.5 ± 0.4	3.985	1	0.050	0.388	1	0.535
PH-C, mean score (T2)	1.5 ± 0.4	1.3 ± 0.3	1.5 ± 0.4	0.523	1	0.472	1.746	1	0.189
Heart rate, bpm (T1)	81.9 ± 11.9	81.5 ± 13.6	79.0 ± 10.1	-	-	-	0.943	1	0.334
Heart rate, bpm (T2)	78.3 ± 13.5	75.9 ± 11.3	72.7 ± 10.0	-	-	-	7.867	1	0.006
Systolic blood pressure, mmHg (T1)	118.6 ± 21.0	119.0 ± 15.7	149.0 ± 29.0	-	-	-	0.341	1	0.561
Systolic blood pressure, mmHg (T2)	118.0 ± 27.2	120.4 ± 18.0	151.2 ± 28.9	-	-	-	1.745	1	0.190
Diastolic blood pressure, mmHg (T1)	70.1 ± 11.5	74.1 ± 11.6	88.5 ± 18.9	-	-	-	1.298	1	0.257
Diastolic blood pressure, mmHg (T2)	74.4 ± 13.1	73.5 ± 9.6	91.2 ± 19.6	-	-	-	.083	1	0.773

### Subjective parameters of anxiety before and after MRI scanning or EEG recording

A descriptive analysis of the frequency distribution of subjective anxiety values (Table 3) yielded the following results: no patient in any of the three sample groups had abnormal values in their answers to a questionnaire about their subjective experience during the procedure, or with respect to physiological arousal (PH-C). With respect to situational anxiety, 39.3% of subjects in the MRI-A group had abnormal values before the procedure, and 29.6% of them had abnormal values after the procedure. The corresponding percentages of abnormal situational anxiety values before and after the procedure were 21.8% and 12.7% in the MRI-C group and 6.6% and 6.3% in the EEG-C group.

**Table 3:** Frequency distribution of the subjective anxiety parameters before (T1) and after (T2) MRI or EEG.

		MRI-C					EEG-C					MRI-A				
**PH-C, mean score**		**1.0–1.9**	**2.0–2.9**	**3.0–3.9**	**4.0–4.9**	**5**	**1.0–1.9**	**2.0–2.9**	**3.0–3.9**	**4.0–4.9**	**5**	**1.0–1.9**	**2.0–2.9**	**3.0–3.9**	**4.0–4.9**	**5**
T1	%	96.5	3.5	-	-	-	97.0	3.5	-	-	-	96.4	3.6	-	-	-
	N	55	2	-	-	-	64	2	-	-	-	27	1	-	-	-
T2	%	94.7	5.3	-	-	-	93.9	4.5	-	-	-	90.6	8.4	-	-	-
	N	54	3	-	-	-	62	3	-	-	-	49	3	-	-	-
**STAI(C)-S, t value**		**<30**	**30–39**	**40–49**	**50–59**	**>60**	**<30**	**30–39**	**40–49**	**50–59**	**>60**	**<30**	**30–39**	**40–49**	**50–59**	**>60**
T1	%	1.8	16.4	25.5	34.5	21.8	1.6	18.0	32.8	41.0	6.6	-	-	25	35.7	39.3
	N	1	9	14	19	12	1	11	20	25	4	-	-	7	10	11
T2	%	1.8	21.8	41.8	21.8	12.7	3.2	28.6	31.7	30.2	6.3	-	7.4	33.3	29.6	29.6
	N	1	12	23	12	7	2	18	20	19	4	-	2	9	8	8
PEQ, total score*		**<1.0**	**1.0–1.9**	**2.0–2.9**	**3.0–3.9**	≥**4**	**<1.0**	**1.0–1.9**	**2.0–2.9**	**3.0–3.9**	≥**4**	**<1.0**	**1.0–1.9**	**2.0–2.9**	**3.0–3.9**	≥**4**
T2	%	89.1	9.1	1.8	-	-	98.5	1.5	-	-	-	76.9	23.1	-	-	-
	N	49	5	1	-	-	65	1	-	-	-	20	6	-	-	-

STAI-S: State-Trait-Anxiety Inventory, state anxiety scale, STAIC-S: State-Trait-Anxiety Inventory for Children, state anxiety scale, PEQ: Patient Experience Questionnaire, PH-C: Physiologcal Hyperarousal Scale for Children.

### Intergroup comparison of MRI-C vs. MRI-A

There were no statistically significant differences between the groups, either before or after bMRI scanning, with respect to either situational anxiety (STAI(C)-S; *p* = 0.262 and *p* = 0.374, respectively) or physiological arousal (PH-C; *p* = 0.050, *p* = 0.472). The heart rate slowed significantly over the course of the procedure in the MRI-A group (*p* = 0.030), but not in the MRI-C group (*p* = 0.058). Systolic blood pressure did not change significantly in either group (*p* = 0.630, *p* = 0.610). Diastolic blood pressure was significantly higher after the procedure in the MRI-C group (*p* = 0.044), but not in the MRI-A group (*p* = 0.154).

### Intergroup comparison of MRI-C vs. EEG-C

The MRI-C and EEG-C groups did not differ significantly from each other either before or after the respective procedure with regard to situational anxiety (STAI(C)-S: *p* = 0.525 and *p* = 0.875, respectively), physiological arousal (PH-C: *p* = 0.53, *p* = 0.189), or systolic (*p* = 0.561, *p* = 0.190) and diastolic blood pressure (*p* = 0.257, *p* = 0.773). There was no group difference in heart rate before the procedure (*p* = 0.334), but the patients in the MRI-C group had a significantly higher heart rate after the examination than the patients in the EEG-C group (*p* = 0.006).

### Prior MRI experience vs. first-ever MRI scan

Children and adolescents undergoing their first-ever MRI scan scored significantly higher with respect to physiological arousal after the scan (PH-C: *p* = 0.029) than those who had prior experience of MRI. There were no further significant differences between these two groups with respect to any of the other subjective or physiological variables (Table 4). In the MRI-C group, participants’ age, sex, IQ, anxiety trait score, number of prior MRI scans, and anxiety about the finding of the MRI scan were no significant predictors of either situational anxiety (STAI(C)-S; adjusted R^2^ = 0.058, F(6, 43) = 1.50, *p* = .201), or physiological arousal (PH-C; adjusted R^2^ = 0.123, F(6, 43) = 2.14, *p* = .068).

**Table 4:** Comparison of children and adolescents undergoing MRI for the first time (N = 23) with children and adolescents with prior MRI experience (N = 34).

Parameters	First-time MRI *M ± SD*	Prior MRI experience *M ± SD*	*t*	*df*	*p*
STAIC-S, t-value (T1)	54.2 ± 11.3	48.6 ± 10.6	1.87	53	0.067
STAIC-S, t-value (T2)	49.1 ± 10.2	44.6 ± 10.2	1.56	53	0.124
PH-C, mean score (T1)	1.4 ± 0.4	1.4 ± 0.4	0.137	54	0.892
PH-C, mean score (T2)	1.6 ± 0.5	1.3 ± 0.3	2.53	55	0.014
Heart rate, bpm (T1)	80.4 ± 10.0	83.8 ± 13.0	-0.99	50	0.328
Heart rate, bpm (T2)	79.3 ± 15.9	80.2 ± 13.9	0.21	49	0.835
Systolic blood pressure, mmHg (T1)	120.1 ± 18.6	118.5 ± 21.5	0.27	50	0.785
Systolic blood pressure, mmHg (T2)	117.4 ± 17.8	118.5 ± 30.4	-0.15	49	0.881
Diastolic blood pressure, mmHg (T1)	70.0 ± 13.7	71.6 ± 10.0	-0.59	50	0.556
Diastolic blood pressure, mmHg (T2)	72.0 ± 10.4	75.2 ± 14.0	-0.87	49	0.390

PH-C: Physical Hyperarousal Scale for Children (25), STAIC-S: State-Trait-Anxiety Inventory for Children, state anxiety scale (26).

## Discussion

The main findings of this study can be summarized as follows:

1. Children and adolescents, as a group, did not differ significantly from adults in any way with respect to anxiety in the setting of bMRI scans.
2. In the Patient Experience Questionnaire, more than 98% of the children and adolescents stated that they had felt little or no anxiety during the bMRI scan itself.
3. The anxiety experienced by children and adolescents undergoing bMRI scanning did not differ from that of children and adolescents undergoing EEG recording.

The central finding of this study–the lack of a difference between children and adolescents on the one hand, and adults on the other, with respect to anxiety experienced during bMRI scanning–is in accordance with the findings of Shechner et al. and Thomason et al. (Table 2; [13, 14]). The relatively low percentage of children and adolescents with marked anxiety during the scan was in the same range as that obtained by Shechner et al. [13] and Marshall et al. [3].

The findings of the present study were obtained in a clinical sample, rather than in a group of persons who were healthy research subjects, as in the studies of Shechner et al. [13] and Thomason et al. (11). This fact hampers comparisons across studies, but also lends additional validity to the findings of the present study, as one can assume that MRI scans performed for clinical purposes would generally be more likely to cause stress than those performed purely for research.

Within the age group of children and adolescents, there was no association between age and anxiety. This finding accords with the majority of previous studies (Table 5).

**Table 5:** Review of previous studies on anxiety experienced by children, adolescents or adults undergoing MRI scanning.

Authors	Country	Study setting	Age (years)	Sample composition	Measurements	Results
**Children and adolescents**						
**Haddad et al. 2013 [10]**	UK	- fMRI for research - Duration: 60 min.	12–18 (*M =* 15.7; *SD =* 1.45)	N = 36 (72.2% females)	- 9-item online questionnaire (subjective experience) at various time points during the diagnostic procedure	- Anxious adolescents showed more anxiety than non-anxious ones - No evidence that anxious adolescents would avoid future MRI scans
**Westra et al. 2011 [9]**	NL	- Two hospitals - Diagnostic MRI of any part of the body (of which N = 19 bMRI, N = 12 with IV access) - Median duration: 20 min., (range: 10–90 min.)	5–12 (median: 9.15)	N = 54 (61.1% females)	- Self-assessment of stress before and after (4-point Likert Scale) - Assessment of anxiety by parent/investigator before and after (5-point scale) - Heart rate (4 time points) - Cortisol in saliva (4 time points) - After MRI scanning: evaluation of MRI compared to blood drawing/vaccination	- No association of age with anxiety - 10% of patients found MRI scanning more unpleasant than blood drawing or vaccination - Positive association of parental trait anxiety with parental assessment of the child’s anxiety - Positive association of contrast medium use and a rise in salivary cortisol
**Tyc et al. 1995 [11]**	USA	- Outpatients of a pediatric oncology service (with a median of 6 prior MRIs) - Diagnostic MRI of any part of the body (of which N = 41 bMRI, N = 13 under sedation)	8.1–21.9 (median: 11.9)	N = 55 (41.8% females)	- Pre: State and Trait Anxiety Inventory (STAI) for parents and child - Post: 15-item questionnaire for parents and child on stress due to MRI scanning	- No association of age with anxiety - Approximately 30% of the children and parents stated that MRI scanning caused significant stress - The placement of an IV catheter was judged by parents and children as the most unpleasant part of the MRI scanning procedure - Parents rated the stress due to the procedure significantly higher than their children did - There was little agreement between parents’ and children’s own assessments of stress - Parents’ and children’s state anxiety was a predictor of stress
**Marshall et al. 1995 [3]**	USA	- Consecutive patients in a children’s hospital - Diagnostic MRI of any part of the body	10–18	N = 85	- Three items (5-point Likert Scale): (1) anxiety at any time with respect to diagnostic or therapeutic procedures, (2) anxiety before MRI scanning, (3) anxiety during MRI scanning), answered on a - After MRI scanning: Assessment of anxiety during scanning by the radiology assistant (5-point Likert Scale)	- No association of age with anxiety - 71% of patients had no anxiety - After MRI significantly less anxiety than before
**Children and adolescents vs. adults**						
**Shechner et al. 2013 [13]**	USA	- fMRI for research (bMRI; anxiety-inducing paradigms)Optional “dry run” in a simulated MRI scanner	12.1 (±2.7) 13.5 (±2.8) 25.6 (±7.2)	N = 325 (87 anxious children, 140 non-anxious children, 98 non-anxious adults)	After MRI scanning: - 6-item questionnaire on feelings and anxiety experienced during the scan - Children/adults: State and Trait Anxiety Inventory (STAI(C)) - Children: Screen for Child Anxiety Related Emotional Disorders- Child Self-Report (SCARED-C) - Parents: Parental version (SCARED-P) - Assessment by a third party: Pediatric Anxiety Rating Scale (PARS)	- Younger age was associated with more anxiety - The three groups did not differ significantly with respect to experienced anxiety - 4% of the subjects had marked anxiety during the MRI scan - Anxious children and adolescents did not experience more anxiety than non-anxious ones - No association of sex with anxiety
**Galván et al. 2012 [15]**	USA	- fMRI for research (bMRI) - Duration: 16 min.	10.6 14.3 17.1 25.5	N = 55 (8–12 years: N = 14; 13–15 years: N = 14; 16–18 years: N = 13; 22–30 years: N = 14)	Self-assessment of anxiety induced by the sight of the MRI scanner before the scan (4-point Likert Scale)	Younger age was associated with more anxiety
**Thomason et al. 2009 [14]**	USA	fMRI for research	N/A	N = 93 (50 children; 43 adults)	After MRI scanning, online questionnaire on six topics (physical well-being, emotional well-being, pleasure, attention, performance on the fMRI paradigm, interaction with investigator)	- No significant difference between children and adults with respect to physical or emotional well-being or performance on the MRI paradigm - Children had significantly more positive responses than adults with respect to attention, pleasure, and interaction with the investigator

As for the comparison of MRI scanning versus EEG recording in children and adolescents, no other studies of this type have ever been carried out to date, therefore our findings cannot be assessed in the context of existing research literature. However, our findings indicate that–in view of the customary assessment of EEG recordings as a minimal burden–because of the similar anxiety levels, MRI scans for research purposes in children and adolescents should be considered a minimal burden as well.

The finding that prior experience of MRI scanning was no predictor for the anxiety experience militates against the hypothesis of habituation through repeated scanning [24], and also, conversely, against the hypothesis of reinforcement of anxiety that may have been induced by earlier MRI scans.

Nonetheless, as shown in Table 2, at least some children and adolescents did, in fact, experience the MRI scan as stressful. It is unclear whether the use of a head coil for bMRI might make this type of scan more prone to induce anxiety than an MRI scan of another part of the body. Also, all participants in our study were examined for diagnostic purposes, which may have caused anxiety regarding the results of the scan, in contrast to the generally healthy volunteers who undergo MRI scanning in research studies.

A further interesting finding is the difference between the reported anxiety levels in the PH-C, and in the STAIC-S. In the PH-C, and also in the PEQ, more than 98% of the children and adolescents reported little or no anxiety; yet the percentage of subjects that reported little or no situational anxiety in the STAIC was markedly lower, at 76–82% (Table 3). One explanation for this may be that the STAIC addressed the situational anxiety associated with the subject’s overall experience in the setting where the study was performed, and not just the anxiety associated with the MRI scan *per se*, as the PEQ and the PH-C did. It is also noteworthy that trait anxiety, as measured by the STAIC, was not a predictor for anxiety during MRI scanning, so this variable does not seem suitable for the pre-procedural identification of patients at risk of experiencing anxiety during the scan. The differences in blood pressure and heart rate before and after MRI scanning, though statistically significant, are clinically irrelevant.

Finally, anxiety during MRI scanning can be lessened by age-appropriate information for children, as well as by distraction [25], but these interventions were not part of the study protocol. Moreover, it should be kept in mind that, for at least some children, MRI scanning is not a source of stress, but actually a positive experience [14, 26, 27].

### Strengths and limitations

One strength of this study is its design with two comparison groups, one consisting of adults and one consisting of children and adolescents, unlike most of the previous studies. The choice of measuring instruments ensured comparability between groups, and anxiety was measured on both the subjective and the objective, physiological level.

The fact that the study was carried out in a routine clinical setting may have led to higher anxiety levels in comparison to MRI scans in a research setting.

It had originally been intended to include only persons who had never previously undergone MRI scanning. Over the course of recruitment of subjects for the study, it became necessary to deviate from this plan, because patients of this type are too rarely encountered in university-affiliated tertiary-care hospitals. A further limitation of this study is the lack of precise standardization of MRI scanning procedures in routine clinical practice.

As for the variables that were assessed in this study, it remains unclear which components of the subjects’ anxiety were registered–that is, whether anxiety was related to MRI scanning *per se*, the clinical setting, or the potential findings of the MRI scan. With respect to the variable “heart rate,” measurement at only two time points (before and after scanning) might not be adequately informative.

In future studies, the use of MRI-specific psychometric instruments (e.g., the MRI-Fear Survey Schedule [28] or the Profile of Mood State [29]) or the inclusion of further physiological measurements (e.g., saliva samples, skin conductivity), might yield additional information.

### Conclusions

In this study, children and adolescents undergoing a brain MRI scan did not experience significantly more anxiety than those undergoing an EEG registration, or adults undergoing brain MRI scanning. Therefore, a general exclusion of minors from MRI research studies does not appear reasonable.
